# Risk of Major Osteoporotic Fracture After Cardiovascular Disease: A Population-Based Cohort Study in Taiwan

**DOI:** 10.2188/jea.JE20120071

**Published:** 2013-03-05

**Authors:** Shih-Wei Lai, Kuan-Fu Liao, Hsueh-Chou Lai, Pang-Yao Tsai, Cheng-Li Lin, Pei-Chun Chen, Fung-Chang Sung

**Affiliations:** 1 School of Medicine, China Medical University, Taichung, Taiwan; 2 Department of Family Medicine, China Medical University Hospital, Taichung, Taiwan; 3 Graduate Institute of Integrated Medicine, China Medical University, Taichung, Taiwan; 4 Department of Internal Medicine, Taichung Tzu Chi General Hospital, Taichung, Taiwan; 5 Department of Health Care Administration, Central Taiwan University of Science and Technology, Taichung, Taiwan; 6 School of Chinese Medicine, China Medical University, Taichung, Taiwan; 7 Department of Internal Medicine, China Medical University Hospital, Taichung, Taiwan; 8 Department of Public Health, China Medical University, Taichung, Taiwan; 9 Management Office for Health Data, China Medical University Hospital, Taichung, Taiwan; 10 Graduate Institute of Epidemiology and Preventive Medicine, College of Public Health, National Taiwan University, Taipei, Taiwan

**Keywords:** atherosclerosis, cardiovascular disease, cerebrovascular disease, fracture, heart failure

## Abstract

**Background:**

We investigated the association between cardiovascular disease (CVD) and the risk of major osteoporotic fracture in Taiwan.

**Methods:**

Using the Taiwan National Health Insurance Database for the period 2000–2007, we classified 43 874 patients aged 50 years or older with newly diagnosed CVD (coronary artery disease, heart failure, cerebrovascular disease, or peripheral atherosclerosis) as the CVD group and 43 874 subjects without CVD (frequency-matched by sex, age, and date selected) as the non-CVD group. Incidence and hazard ratios (HRs) for major osteoporotic fracture of the spine, hip, humerus, and forearm/wrist were estimated for the period until the end of 2010.

**Results:**

After adjustment for confounders, the overall HRs for major osteoporotic fracture were 1.24 (95% CI = 1.13, 1.36) in men with CVD and 1.18 (95% CI = 1.11, 1.25) in women with CVD, as compared with the non-CVD group. As compared with the non-CVD group, the adjusted HR for major osteoporotic fracture was highest among subjects with cerebrovascular disease (HR 1.31; 95% CI 1.23, 1.39), followed by those with heart failure (HR 1.18; 95% CI 1.11, 1.27), peripheral atherosclerosis (HR 1.12; 95% CI 1.04, 1.20), and coronary artery disease (HR 1.07; 95% CI 1.01, 1.12).

**Conclusions:**

CVD is associated with risk of major osteoporotic fracture in men and women in Taiwan.

## INTRODUCTION

Cardiovascular disease (CVD) and osteoporosis are major diseases that cause marked morbidity, disability, and mortality, and a very large socioeconomic burden worldwide.^1^^–^^3^ In their review, Deaton et al noted that CVD accounts for about one-third of all deaths in the world.^1^ Another review showed that osteoporosis is responsible for fractures in about 2 million people in the United States annually.^2^ Recently, accumulating evidence shows a robust association between CVD and osteoporosis/osteoporotic fracture, which share risk factors and pathophysiologic pathways.^4^^–^^9^ Aging, menopause, and chronic inflammation may partially explain this association.^6^^,^^7^ A study in Australia by Chen et al found that fracture was 1.23 times (95% CI = 1.13–1.35) as likely among women with CVD than among those without CVD.^8^ Gerber et al reported that US patients had a 1.32-fold risk of fracture (95% CI = 1.12–1.56) after myocardial infarction.^9^

In 2010, CVD was the second leading cause of death in Taiwan, accounting for 10.8% of all deaths.^10^ In a study using data from the Taiwan National Health Insurance database, Yang et al estimated that the average prevalence of osteoporosis in 1999–2001 was 11.4% in women, and 1.6% in men, aged 50 years or older.^11^ To date, most studies of the association between CVD and osteoporosis/osteoporotic fracture were limited to white populations, and little evidence is available in Taiwan. Identifying risk factors for fracture is important in preventing development of fractures and improving outcomes. In this population-based cohort study, we used claims data from the National Health Insurance program in Taiwan to investigate the association between CVD and risk of major osteoporotic fracture.

## METHODS

### Data sources

This study used claims data from the National Health Insurance program in Taiwan, which has been described in detail in previous studies.^12^^–^^16^ In brief, the National Health Research Institute in Taiwan has used insurance claims data to establish several longitudinal data files on medical services provided to inpatients and outpatients. All data files were linked with scrambled identifiers to protect patient privacy. Disease diagnoses were coded according to the International Classification of Diseases 9th Revision-Clinical Modification (ICD-9-CM). In addition, the A-code was used to define diseases, because its use predated adoption of ICD-9 coding in Taiwan.

### Design

We used claims data covering 1 million insured persons and established 2 study groups. The CVD group included patients who received new diagnoses during 2000–2007 of coronary artery disease (ICD-9 410–414; A-code A270, A279), heart failure (ICD-9 428), cerebrovascular disease (ICD-9 430–438; A-code A290–A294, A299), or peripheral atherosclerosis (ICD-9 440–448; A-code A300). All people younger than 50 years on the day of diagnosis were excluded. For each selected CVD patient, 1 adult without medical claims for CVD was randomly selected for the non-CVD group, after frequency-matching for sex, age (every 5-year span), and index data. The index date was defined as the date of diagnosis for CVD patients and as the middle date of the same index month as their matched CVD patients for non-CVD subjects. To decrease confounding effects, subjects previously diagnosed with fracture at any site (ICD-9 800–829, E887, and A-code A470–A476, A479) before the index date were excluded from the study. Diagnosis of a major osteoporotic fracture, including fracture of the spine (ICD-9 805 and 806), hip (ICD-9 820), humerus (ICD-9 812), and forearm (ICD-9 813)/wrist (ICD-9 814), were used as the study end-point. These fracture sites have been defined by the World Health Organization fracture risk assessment tool, FRAX.^17^ Both the CVD and non-CVD groups were followed up to determine fracture incidence until a subject received a fracture diagnosis, death, withdrawal from the insurance program, loss to follow-up, or December 31, 2010.

Comorbidities potentially associated with major osteoporotic fracture before the index date were hypertension, arrhythmia, hyperlipidemia, obesity, diabetes mellitus, dementia, Parkinson’s disease, depression, chronic kidney disease, osteoporosis, menopause, cancer, thyrotoxicosis, tobacco use, and alcoholism. All were diagnosed by using ICD-9 code and A-code.

### Definition of exposure

Medical therapy for osteoporosis was defined as at least 1 recorded prescription for bisphosphonate, calcitonin, estrogen, or raloxifene before the index date. Use of other bone-related medications was defined as at least 1 prescription for thiazolidinedione, proton-pump inhibitor, glucocorticoid, statin, warfarin, or heparin before the index date.

### Statistical analysis

Demographic status, comorbidities, and medications were compared between the CVD and non-CVD groups among men and women separately. The chi-square test and Student *t* test were used to compare differences between the CVD and non-CVD groups in sociodemographic characteristics, comorbidities, and medications. The incidence rate of fracture was calculated as number of fracture cases identified during follow-up divided by total person-years for each group. Cox proportional hazard models were used to estimate hazard ratios (HRs) and 95% CIs for sex-specific risk of hip fracture and major osteoporotic fracture in association with CVD. Subanalysis evaluated whether different types of CVD were associated with risk of major osteoporotic fracture. All analyses were performed using SAS software version 9.1 (SAS Institute Inc., Cary, NC, USA), and the statistical significance level was defined as a 2-sided *P* value less than 0.05.

## RESULTS

### Baseline characteristics of the study population

Analysis of claims data from 2000–2007 revealed 43 874 patients with CVD (CVD group) and 43 874 subjects free from disease (non-CVD group) who met the eligibility criteria. Men in the CVD group had a significantly shorter mean duration of follow-up than did men in the non-CVD group (6.03 vs 6.44 years, *P* < 0.0001; Table 1). Comorbidities and medications were more frequent in the CVD group than in the non-CVD group, with the exception of obesity, cancer, and use of bisphosphonate and raloxifene. Women in the CVD group had a significantly shorter mean duration of follow-up than did women in the non-CVD group (6.25 vs 6.64 years, *P* < 0.0001; Table 1). As with men, most comorbidities and medications were more frequent in the CVD group than in the non-CVD group, with the exception of obesity, cancer, tobacco use, alcoholism, and use of bisphosphonate and raloxifene. There was no significant difference in medical therapy for osteoporosis between the CVD group and non-CVD group in either sex (data not shown).

**Table 1.** Baseline characteristics of subjects with and without cardiovascular disease

	Men					Women				
	
	Cardiovascular disease					Cardiovascular disease				
	
	No		Yes			No		Yes		
	*N* = 23 161		*N* = 23 161			*N* = 20 713		*N* = 20 713		
			
	*n*	%	*n*	%	*P* value	*n*	%	*n*	%	*P* value
Age group (years)										
50–64	10 017	43.3	9849	42.5	0.11	9785	47.2	9495	45.8	0.004
65–84	13 144	56.8	13 312	57.5		10 928	52.8	11 218	54.2	
Mean (SD) (years)^a^	65.8	9.14	66	9.13	0.08	65	8.91	65.2	8.9	0.028
Follow-up years, mean (SD)	6.44	2.79	6.03	3.01	<0.0001	6.64	2.78	6.25	2.97	<0.0001
Comorbidities before index date										
Hypertension	7818	33.8	16 655	71.9	<0.0001	8050	38.9	15 344	74.1	<0.0001
Arrhythmia	1063	4.59	3747	16.2	<0.0001	1259	6.08	3921	18.9	<0.0001
Hyperlipidemia	1815	7.84	4232	18.3	<0.0001	2539	12.3	4888	23.6	<0.0001
Obesity	11	0.05	19	0.08	0.14	18	0.09	29	0.14	0.11
Diabetes mellitus	3213	13.9	6461	27.9	<0.0001	3233	15.6	6529	31.5	<0.0001
Dementia	179	0.77	377	1.63	<0.0001	140	0.68	292	1.41	<0.0001
Parkinson’s disease	246	1.06	465	2.01	<0.0001	194	0.94	358	1.73	<0.0001
Depression	558	2.41	862	3.72	<0.0001	734	3.54	1269	6.13	<0.0001
Chronic kidney disease	432	1.87	972	4.2	<0.0001	252	1.22	772	3.73	<0.0001
Osteoporosis	390	1.68	628	2.71	<0.0001	2804	13.5	3850	18.6	<0.0001
Menopause	—	—	—	—	—	3905	18.9	4992	24.1	<0.0001
Cancers	894	3.86	785	3.39	0.007	829	4	719	3.47	0.004
Thyrotoxicosis	94	0.41	123	0.53	0.049	267	1.29	382	1.84	<0.0001
Tobacco use	39	0.17	80	0.35	0.0002	4	0.02	6	0.03	0.53
Alcoholism	55	0.24	107	0.46	<0.0001	3	0.01	6	0.03	0.32
Ever use of medications										
Thiazolidinedione	260	1.12	564	2.44	<0.0001	216	1.04	623	3.01	<0.0001
Proton-pump inhibitor	999	4.31	1746	7.54	<0.0001	569	2.75	1087	5.25	<0.0001
Glucocorticoid	3509	37.6	19 652	53.1	<0.0001	2043	35.4	18 670	52.4	<0.0001
Statin	1031	4.45	3006	13	<0.0001	1533	7.4	3407	16.5	<0.0001
Warfarin	87	0.38	551	2.38	<0.0001	55	0.27	374	1.81	<0.0001
Heparin	209	0.9	1454	6.28	<0.0001	166	0.8	718	3.47	<0.0001
Bisphosphonate	13	0.06	17	0.07	0.47	109	0.53	119	0.57	0.51
Calcitonin	30	0.13	62	0.27	0.0008	153	0.74	224	1.08	0.0002
Estrogen	—	—	—	—	—	1652	7.98	2051	9.9	<0.0001
Raloxifene	1	0	0	0	0.99	25	0.12	25	0.12	0.99

The chi-square test and ^a^*t*-test were used to compare subjects with and without cardiovascular disease.

### Association between CVD and hip fracture

Table 2 shows the sex-specific risk of hip fracture by duration of follow-up for the CVD and non-CVD groups. The incidence of hip fracture was highest among women with CVD (4.27 per 1000 person-years), followed by women without CVD, men with CVD, and men without CVD (1.96 per 1000 person-years). Multivariate Cox regression analysis revealed that the HR for hip fracture associated with CVD was not significant (HR, 1.02; 95% CI = 0.92, 1.13).

**Table 2.** Incidence and hazard ratios (HRs) for hip fracture among subjects with and without cardiovascular disease (CVD), by sex and duration of follow-up

	Non-CVD			CVD			Crude HR (95% CI)	Adjusted HR (95% CI)
	
	Events	Person-years	Incidence rate^a^	Events	Person-years	Incidence rate^a^		
Men	298	151 808	1.96	399	142 873	2.79	1.43 (1.23, 1.66)	0.93 (0.79, 1.10)
Follow-up years								
≤1	32	22 793	1.40	46	22 186	2.07	1.47 (0.94, 2.32)	0.77 (0.45, 1.32)
>1	266	129 016	2.06	353	120 686	2.92	1.42 (1.21, 1.67)	0.95 (0.79, 1.13)

Women	422	145 104	2.91	591	138 555	4.27	1.47 (1.30, 1.67)	1.11 (0.97, 1.27)
Follow-up years								
≤1	36	20 528	1.75	76	20 146	3.77	2.15 (1.45, 3.20)	0.96 (0.63, 1.45)
>1	386	124 577	3.10	515	118 409	4.35	1.41 (1.24, 1.61)	1.13 (0.98, 1.30)

Overall	720	296 913	2.42	990	281 428	3.52	1.46 (1.32, 1.60)	1.02 (0.92, 1.13)
Follow-up years								
≤1	68	43 320	1.57	122	42 333	2.88	1.84 (1.36, 2.47)	0.87 (0.63, 1.21)
>1	652	253 592	2.57	868	239 096	3.63	1.42 (1.28, 1.57)	1.03 (0.93, 1.16)

Adjusted HR in men: adjusted for age, hypertension, arrhythmia, diabetes mellitus, dementia, Parkinson’s disease, depression, chronic kidney disease, osteoporosis, alcoholism, proton-pump inhibitor, glucocorticoid, and calcitonin.

Adjusted HR in women: adjusted for age, hypertension, arrhythmia, hyperlipidemia, diabetes mellitus, dementia, Parkinson’s disease, chronic kidney disease, osteoporosis, menopause, thiazolidinedione, glucocorticoid, bisphosphonate, calcitonin, estrogen, and raloxifene.

Adjusted overall HR: adjusted for age, diabetes mellitus, hyperlipidemia, Parkinson’s disease, osteoporosis, glucocorticoid, bisphosphonate, calcitonin, and estrogen.

^a^Incidence rate: per 1000 person-years.

### Association between CVD and major osteoporotic fractures

Table 3 shows the sex-specific risk of major osteoporotic fracture by duration of follow-up among adults with and without CVD. The incidence of fracture was highest among women with CVD (21.5 per 1000 person-years), followed by women without CVD, men with CVD, and men without CVD (7.02 per 1000 person-years). Multivariate Cox regression analysis revealed that the HR for major osteoporotic fracture associated with CVD was 1.16 (95% CI 1.10, 1.22). The sex-specific HRs were similar for men and women (HR, 1.24 vs 1.18). The risk of fracture declined slightly after 1 year of follow-up.

**Table 3.** Incidence and hazard ratios (HRs) for major osteoporotic fractures among subjects with and without cardiovascular disease (CVD), by sex and duration of follow-up

	Non-CVD			CVD			Crude HR (95% CI)	Adjusted HR (95% CI)
	
	Events	Person-years	Incidence rate^a^	Events	Person-years	Incidence rate^a^		
Men	1047	149 200	7.02	1276	139 562	9.14	1.31 (1.20, 1.42)	1.24 (1.13, 1.36)
Follow-up years								
≤1	128	22 747	5.63	195	22 109	8.82	1.57 (1.25, 1.96)	1.47 (1.15, 1.88)
>1	919	126 453	7.27	1081	117 453	9.20	1.27 (1.16, 1.39)	1.21 (1.09, 1.33)

Women	2324	137 578	16.9	2786	129 484	21.5	1.28 (1.21, 1.35)	1.18 (1.11, 1.25)
Follow-up years								
≤1	296	20 404	14.5	412	19 972	20.6	1.42 (1.23, 1.65)	1.32 (1.12, 1.55)
>1	2028	117 174	17.3	2374	109 512	21.7	1.26 (1.18, 1.33)	1.16 (1.08, 1.23)

Overall	3371	286 778	11.8	4062	269 047	15.1	1.29 (1.23, 1.35)	1.16 (1.10, 1.22)
Follow-up years								
≤1	424	43 152	9.83	607	42 081	14.4	1.47 (1.30, 1.66)	1.33 (1.16, 1.52)
>1	2947	243 627	12.1	3455	226 965	15.2	1.26 (1.20, 1.32)	1.14 (1.08, 1.20)

Adjusted HR in men: adjusted for age, hypertension, arrhythmia, diabetes mellitus, dementia, Parkinson’s disease, depression, chronic kidney disease, osteoporosis, alcoholism, proton-pump inhibitor, glucocorticoid, and calcitonin.

Adjusted HR in women: adjusted for age, hypertension, arrhythmia, hyperlipidemia, diabetes mellitus, dementia, Parkinson’s disease, chronic kidney disease, osteoporosis, menopause, thiazolidinedione, glucocorticoid, bisphosphonate, calcitonin, estrogen, and raloxifene.

Adjusted overall HR: adjusted for age, diabetes mellitus, hyperlipidemia, Parkinson’s disease, osteoporosis, glucocorticoid, bisphosphonate, calcitonin, and estrogen.

^a^Incidence rate: per 1000 person-years.

### Subanalysis of the association between type of CVD and major osteoporotic fracture

The association between type of CVD and risk of major osteoporotic fracture was also analyzed (Table 4). As compared with the non-CVD group, the adjusted HR of major osteoporotic fracture was highest among patients with cerebrovascular disease (HR 1.31, 95% CI 1.23, 1.39), followed by those with heart failure (1.18; 1.11, 1.27), peripheral atherosclerosis (1.12; 1.04, 1.20), and coronary artery disease (1.07; 1.01, 1.12).

**Table 4.** Hazard ratios (HRs) and 95% CIs for major osteoporotic fracture associated with 4 types of cardiovascular disease (CVD)

	Crude HR (95% CI)	Adjusted HR (95% CI)
Men		
Non-CVD (reference)	1.00 (Reference)	1.00 (Reference)
Coronary artery disease	1.09 (1.00, 1.18)	1.05 (0.96, 1.15)
Heart failure	1.58 (1.41, 1.78)	1.29 (1.14, 1.45)
Cerebrovascular disease	1.71 (1.55, 1.89)	1.47 (1.33, 1.63)
Peripheral atherosclerosis	1.32 (1.16, 1.50)	1.21 (1.06, 1.37)
Women		
Non-CVD (reference)	1.00 (Reference)	1.00 (Reference)
Coronary artery disease	1.16 (1.10, 1.23)	1.12 (1.05, 1.18)
Heart failure	1.45 (1.34, 1.58)	1.12 (1.03, 1.22)
Cerebrovascular disease	1.67 (1.55, 1.80)	1.32 (1.23, 1.43)
Peripheral atherosclerosis	1.16 (1.06, 1.26)	1.10 (1.01, 1.20)
Overall		
Non-CVD (reference)	1.00 (Reference)	1.00 (Reference)
Coronary artery disease	1.15 (1.10, 1.20)	1.07 (1.01, 1.12)
Heart failure	1.50 (1.40, 1.60)	1.18 (1.11, 1.27)
Cerebrovascular disease	1.57 (1.48, 1.66)	1.31 (1.23, 1.39)
Peripheral atherosclerosis	1.23 (1.15, 1.32)	1.12 (1.04, 1.20)

Adjusted HR in men: adjusted for age, hypertension, arrhythmia, diabetes mellitus, dementia, Parkinson’s disease, depression, chronic kidney disease, osteoporosis, alcoholism, proton-pump inhibitor, glucocorticoid, and calcitonin.

Adjusted HR in women: adjusted for age, hypertension, arrhythmia, hyperlipidemia, diabetes mellitus, dementia, Parkinson’s disease, chronic kidney disease, osteoporosis, menopause, thiazolidinedione, glucocorticoid, bisphosphonate, calcitonin, estrogen, and raloxifene.

Adjusted overall HR: adjusted for age, diabetes mellitus, hyperlipidemia, Parkinson’s disease, osteoporosis, glucocorticoid, bisphosphonate, calcitonin, and estrogen.

## DISCUSSION

Although this study is not novel, it is to our knowledge the first population-based cohort study to examine the association between CVD and risk of major osteoporotic fracture in Taiwan. We selected only adults aged 50 years or older, and the prevalences of most comorbidities and medications were therefore greater among the CVD group than among the non-CVD group. The incidence of major osteoporotic fracture among women was more than 2-fold that among men, regardless of CVD status. Our finding of a risk of major osteoporotic fracture was somewhat comparable to the results of previous studies (risk rates, 1.2 to 1.5).^7^^–^^9^ The present study found that the overall risk of fracture in the CVD group was approximately 1.2-fold that of the non-CVD group. However, there were variations in men and women with regard to duration of follow-up and interaction between comorbidities.

Additional analysis by CVD type showed that cerebrovascular disease (HR 1.31), heart failure (HR 1.18), peripheral atherosclerosis group (HR 1.12), and coronary artery disease (HR 1.07) were substantially associated with increased risk of major osteoporotic fracture. Two studies by Sennerby et al showed that Swedish patients with cerebrovascular disease had higher fracture risk (OR = 2.76 and HR = 5.09),^4^^,^^5^ and our findings are similar. The authors suggested that the increased likelihood of falling after cerebrovascular disease partially explained this trend.^4^^,^^5^

Variation in fracture risk during follow-up is important in preventive care. Though the absolute incidence rate of major osteoporotic fracture was higher after 1 year of follow-up, the hazard was lower during subsequent follow-up. In contrast, Sennerby et al found a higher risk of hip fracture within the first year after a CVD diagnosis in Sweden.^4^^,^^5^ In the present study, the absolute incidence rate of hip fracture was higher among the CVD group, but the HR was not statistically significant (HR 1.02, 95% CI 0.92, 1.13). Although the time-course effect differed between previous studies and the present study, we believe that clinicians should be mindful of the risk of major osteoporotic fracture even at 1 year after a CVD diagnosis, as risk did not substantially vary during the follow-up period.

Some limitations should be addressed. First, because claims data were used, some CVD diagnoses might not fulfill all criteria for each disease. Also, instances of fracture at hip or major skeletal sites could not be diagnosed as fragility fracture. This limitation may have led to underestimation of fracture risk. Second, because claims data did not provide information on bone mineral density (BMD), we were unable to evaluate fracture risk in relation to presence of osteoporosis, osteopenia, or normal BMD. Therefore, we were unable to examine the relation between CVD and BMD. Third, although there was no record of body mass index (BMI) in the claims data, we were able to include information on obesity (ie, ICD-9 278.00 and 278.01 and A-code A183) as a comorbidity in the analysis.

### Conclusion

After careful adjustment for covariates and consideration of duration of follow-up, CVD was associated with increased risk of major osteoporotic fracture in both sexes, although the incidence of such fractures was higher among women than among men.
